# Effect of anticoagulation on the incidence of venous thromboembolism, major bleeding, and mortality among hospitalized COVID-19 patients: an updated meta-analysis

**DOI:** 10.3389/fcvm.2024.1381408

**Published:** 2024-04-05

**Authors:** Xinwang Chen, Suyun Zhang, Haiyu Liu, Qianyuan Zhang, Jinghan Chen, Qixian Zheng, Ningjing Guo, Yuanyuan Cai, Qiong Luo, Qian Xu, Sheng Yang, Xiangqi Chen

**Affiliations:** ^1^ Department of Pulmonary and Critical Care Medicine, Fujian Medical University Union Hospital, Fuzhou, China; ^2^ Department of Internal Medicine, Fujian Medical University Union Hospital, Fuzhou, China; ^3^ Department of General Medicine, Fujian Medical University Union Hospital, Fuzhou, China; ^4^ Department of Oncology Medicine, Fujian Medical University Union Hospital, Fuzhou, China; ^5^ Fujian Key Laboratory of Translational Research in Cancer and Neurodegenerative Diseases, Fuzhou, China

**Keywords:** venous thromboembolism, COVID-19, thromboprophylaxis, anticoagulation, prevention and treatment

## Abstract

**Objective:**

Anticoagulation is crucial for patients hospitalized with coronavirus disease 2019 (COVID-19) due to the high risk of venous thromboembolism (VTE). However, the optimal anticoagulation regimen needs further exploration. Therefore, we evaluated the efficacy and safety of diverse anticoagulation dosage dosages for COVID-19.

**Methods:**

An updated meta-analysis was performed to assess the effect of thromboprophylaxis (standard, intermediate, and therapeutic dose) on the incidence of VTE, mortality and major bleeding among COVID-19 patients. Literature was searched via PubMed, EMBASE, Web of Science, and China National Knowledge Infrastructure (CNKI) database. The odds ratio (OR) and 95% confidence interval (CI) were calculated for effect estimates.

**Results:**

Nineteen studies involving 25,289 participants without VTE history were included. The mean age of patients was 59.3 years old. About 50.96% were admitted to the intensive care unit. In the pooled analysis, both therapeutic-dose and intermediate-dose anticoagulation did not have a significant advantage in reducing VTE risk over standard dosage (OR = 1.09, 95% CI: 0.58–2.02, and OR = 0.89, 95% CI: 0.70–1.12, respectively). Similarly, all-cause mortality was not further decreased in either therapeutic-dose group (OR = 1.12, 95% CI: 0.75–1.67) or intermediate-dose group (OR = 1.34, 95% CI: 0.83–2.17). While the major bleeding risk was significantly elevated in the therapeutic-dose group (OR = 2.59, 95%CI: 1.87–3.57) as compared with the standard-dose regimen. Compared with intermediate dosage, therapeutic anticoagulation did not reduce consequent VTE risk (OR = 0.85, 95% CI: 0.52–1.38) and all-cause mortality (OR = 0.84, 95% CI: 0.60–1.17), but significantly increased major bleeding rate (OR = 2.42, 95% CI: 1.58–3.70). In subgroup analysis of patients older than 65 years, therapeutic anticoagulation significantly lowered the incidence of VTE in comparation comparison with standard thromboprophylaxis, however, at the cost of elevated risk of major bleeding.

**Conclusion:**

Our results indicated that for most hospitalized patients with COVID-19, standard-dose prophylactic anticoagulation might be the optimal choice. For elderly patients at low risk of bleeding, therapeutic-dose anticoagulation could further reduce VTE risk and should be considered especially when there were other strong risk factors of VTE during hospital stay.

**Systematic Review Registration:**

https://www.crd.york.ac.uk/PROSPERO, identifier, CRD42023388429.

## 1 Introduction

Since December 2019, the coronavirus disease 2019 (COVID-19) pandemic, caused by severe acute respiratory syndrome coronavirus 2 (SARS-CoV-2), has led to large-scale human transmission and caused hundreds of thousands deaths around the world (1). Due to complexity and heterogenous severity of COVID-19, large difficulties and challenges in disease management have been brought by its complications during clinical practice, among which venous thromboembolism (VTE) deserves more attention being paid to because of potential fatal events, especially in early pandemic era (2). VTE includes deep vein thrombosis (DVT) and pulmonary embolism (PE). As is known, PE is caused by an obstruction of the pulmonary arteries, most often occluded by thrombus derived from DVT of the lower extremities, and its major symptoms include dyspnea, chest pain, syncope, hemoptysis, etc. (3) and dyspnea. In patients with COVID-19, significant abnormalities in coagulation function have been reported (4). In addition to this, vascular wall injuries, blood stream stasis, and hypercoagulable state in hospitalized COVID-19 patients increases the risk of VTE (5–7). Unpredictable deterioration and even sudden death may occur in some COVID-19 patients due to secondary VTE event during disease management (8). Thus, early recognition of risk factors and appropriate thromboprophylaxis of VTE in patients with COVID-19 are crucial for lowering in-hospital mortality and may to improve long-term prognosis.

Growing evidence has shown that prophylactic anticoagulation can effectively reduce the incidence of VTE and mortality rate in hospitalized COVID-19 patients with COVID-19, especially in critically ill patients, although at price of increased risk of bleeding (9–11). However, various dosages of prophylactic anticoagulation are used in practice to balance clinical benefit and bleeding risk. Still, no valid consensus has been reached regarding optimal anticoagulation dosage for VTE prevention in COVID-19 patients to achieve best efficacy and less hemorrhage event (12–16).

Although previous meta-analysis has addressed this issue, emerging new studies with diverse outcomes have been published later, and various virus strain of SARS-CoV-2 has evolved which may possess different impact on VTE risk.

Therefore, we conducted this updated meta-analysis to evaluate the efficacy and safety of different prophylactic anticoagulation regimen (standard dose, intermediate dose, and therapeutic dose) on the incidence of VTE, major bleeding, and mortality, to obtain better and more detailed evidence on VTE prophylaxis for hospitalized patients with COVID-19.

## 2 Methods

### 2.1 Design

Low molecular weight heparins are most frequently used for thromboprophylaxis in COVID-19. Therefore, in this meta-analysis, we assessed three conventional prophylactic anticoagulation regimen with low molecular weight heparins (shown in Table 1) on the incidence of VTE, major bleeding, and mortality among COVID-19 patients. This systematic review and meta-analysis was reported in accordance with the Cochrane Handbook (17) and the guidance from Preferred Reporting Items of Systematic Reviews and Meta-Analyses (PRISMA) checklist (18). The protocol of this study has been registered on the International Prospective Register of Systematic Reviews (PROSPERO, https://www.crd.york.ac.uk/PROSPERO) with registration number of CRD42023388429.

**Table 1:** Doses of low molecular weight heparin administered in the three anticoagulation regimens.

	Prophylactic dose		Intermediate dose		Therapeutic dose	
	CrCl>30 ml/min	CrCl ≤30 ml/min	CrCl>30 ml/min	CrCl ≤30 ml/min	CrCl>30 ml/min	CrCl ≤30 ml/min
Enoxaparin	40 mg/24 h	20 mg/24 h	1 mg/kg/24 h >80 kg:60 mg/24 h	0.5 mg/kg/24 h >80 kg:40 mg/24 h	1.5 mg/kg/24 h or 1 mg/kg/12 h	1 mg/kg/24 h
Tinzaparin	4,500 IU/24 h	4,500 IU/24 h	75 IU/kg/24 h >90 kg:50 IU/kg/24 h	75 IU/kg/24 h >90 kg:50 IU/kg/24 h	175 IU/kg/24 h	175 IU/kg/24 h
Bemiparin	3,500 IU/24 h	2,500 IU/24 h	5,000 IU/24 h	3,500 IU/24 h	115 IU/kg/24 h	85 IU/kg/24 h
Fondaparinux	2.5 mg/24 h	1.5 mg/24 h	5 mg/24 h	2.5 mg/24 h	<50 kg: 5 mg/24 h 51–100 kg: 7.5 mg/24 h >100 kg: 10 mg/24 h	Not recommended

Mg, milligrams; IU, international units; kg, kilograms; h, hours; CrCl, calculated creatinine clearance rate.

### 2.2 Search strategy

We designed a high-sensitivity search strategy that combined the following search items: free-text and keyword synonyms of COVID-19 and VTE, and word clusters of prophylactic anticoagulation. Literature was searched through PubMed, EMBASE, Web of Science, and China National Knowledge Infrastructure (CNKI) database. We further searched with the keywords “standard-dose prophylactic anticoagulation”, “intermediate-dose prophylactic anticoagulation”, and “therapeutic-dose prophylactic anticoagulation” on bioRxiv (http://www.biorxiv.org) server, medRxiv (http://www.biorxiv.org) server and Chinaxiv (http://biotech.chinaxiv.org) server, in order to identify potential pre-publication manuscripts that met the eligibility criteria. The search spanned from January 1, 2020to October 31, 2022. The reference lists of all included articles were also reviewed for potential eligible studies.

### 2.3 Study selection and data extraction

Two reviewers independently performed a two-step selection, screening by title and abstract, followed by a full-text review. Studies would be included if they met the following criteria: (1) they were randomized controlled trial, observational cohort, or case-control study; (2) they enrolled hospitalized COVID-19 patients without VTE at baseline who did not receive anticoagulation in the past six months; (3) outcomes of interest were compared among patients receiving standard-dose prophylactic anticoagulation, intermediate-dose prophylactic anticoagulation and therapeutic-dose prophylactic anticoagulation; (4) outcomes of interest included one of the followings: event of VTE, major bleeding, and mortality.

Exclusion criteria were as follows: (1) non-human studies; (2) non-comparative studies; (3) studies that did not recruit COVID-19 patients; (4) studies with no available data to extract; (5) certain type of studies like reviews, meta-analysis, or editorials.

Data extraction was conducted using standardized data extraction forms. The following information were collected from the retrieved literature: the first author's name, publication year, study design, research site, patient characteristics (including age, gender, and disease severity), follow-up period, incidence of VTE, major bleeding, and mortality rate. Discrepancies were solved by discussion.

### 2.4 Statistical analysis

Effects of 3 different dosing prophylactic anticoagulation on the incidence of VTE, major bleeding, and mortality of COVID-19 patients were presented or calculated as odds ratio (OR), relative risk (RR), or hazard ratio (HR), with 95% confidence interval (CI) from included studies. We pooled ORs across studies using inverse-variance weighted DerSimonian-Laird method to calculate effect estimate. RR and HR were considered as equivalent as OR during meta-analysis. Continuous variables were calculated as weighted mean difference (WMD) and 95% CI. The median value and interquartile range (IQR) provided from original studies were converted to mean and standard deviation (SD) according to the method by Wan et al. (19). Between-study heterogeneity was tested by Cochrane Q and I^2^ statistic. I^2^ > 50% or *P *< 0.1 was considered as significant heterogeneity and random effects model was used to combine the results. Otherwise, fixed effects model was used (17). Funnel plots and sensitivity analysis were then conducted to examine the publication bias and stability of meta-analysis result, respectively. All statistical analysis process were conducted using Review Manager 5.4 (Cochrane Collaboration, Oxford, UK) and STATA 14.0 (Stata Corporation, College Station, TX, U.S).

### 2.5 Literature quality evaluation

The methodological quality of included articles was evaluated using the Newcastle-Ottawa Scale (NOS), available at: https://www.ohri.ca/programs/CIinical_epidemiology/oxford.asp. The total score of NOS rangeed from 0 to 9 stars, with more stars representing higher quality. Two authors independently went through this scoring process, and discrepancies were solved by discussion.

### 2.6 Network meta-analysis

So far, current studies mainly compared the efficacy and safety between intermediate-dose and standard-dose anticoagulation, or between therapeutic-dose and standard-dose anticoagulation for COVID-19 patients. Fewer studies [Jonmarker et al. (20) and Blondon et al. (21)] investigated the difference between intermediate-dose with therapeutic-dose prophylactic anticoagulation, making it less convincing to perform traditional meta-analysis. Thus, we chose network meta-analysis and defined standard-dose anticoagulation as plan A (plan 1 in the rank), intermediate-dose anticoagulation as plan B (plan 2 in the rank), and therapeutic-dose anticoagulation as plan C (plan 3 in the rank). The network meta-analysis was conducted using “mvmeta” package and “network” package of STATA 14.0 software.

## 3 Results

**Figure 1:**
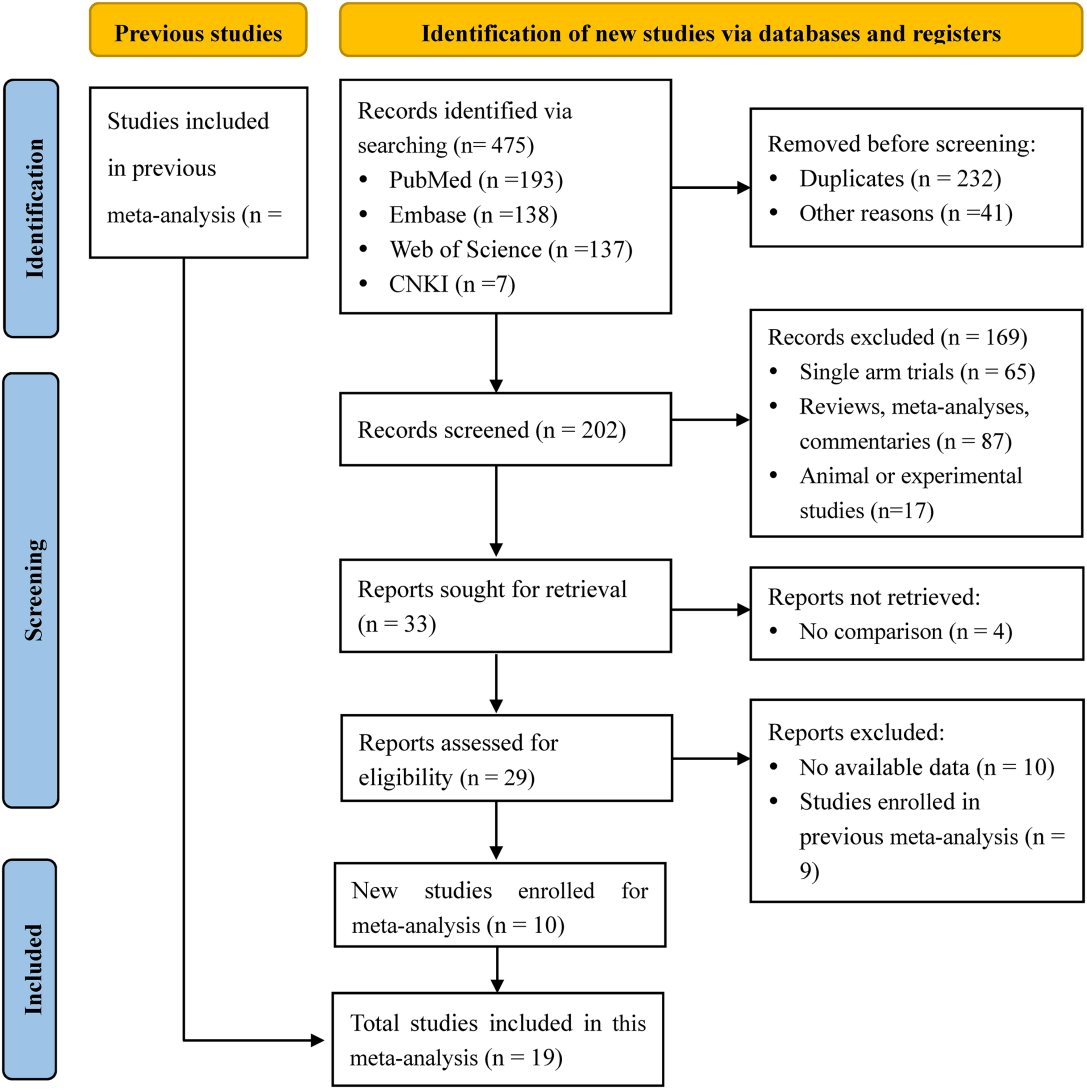
PRISMA flow chart of literature research and selection for updated meta-analysis.

We searched 205 studies and screened 202 studies by title and abstract, and then obtained 33 eligible studies. There were 14 studies excluded after the full-text screening, and we finally included 19 works of literature (10, 12, 16, 22–37) for meta-analysis, containing 3 retrospective cohort studies and 16 randomized controlled trials. Study selection and characteristics were shown in Figure 1 and Table 2, separately. The synthesis results of the meta-analysis were the comprehensive impact of different doses (therapeutic-dose vs. standard-dose, intermediate-dose vs. standard-dose, therapeutic-dose vs. intermediate-dose) of prophylactic anticoagulation on the incidence of VTE, major bleeding, and mortality among COVID-19 patients without VTE at admission.

**Table 2:** Basic characteristics of the included studies.

Study	Country	Groups	Design	No of participants	Age, years, (median, mean ± SD or IQR)	Gender: male, %	Follow-up period	Severe case, %	VTE (event/total)	Major bleeding (event/total)	Mortality (event/total)
Spyropoulos et al. (16)	U.S.	TD vs. SD	Randomized clinical trial, multicenter	253	TD: 65.8 ± 13.9	TD: 52.7	371 days	32.81	TD: 14/129	TD: 6/129	TD: 25/129
					SD: 67.7 ± 14.1	SD: 54.8			SD: 36/124	SD: 2/124	SD: 31/124
Sadeghipour et al. (22)	Iran	ID vs. SD	Randomized clinical trial, multicenter	562	ID: 62 (51.0, 70.7)	ID: 58.7	113 days	100	ID: 9/276	ID: 7/276	ID: 119/276
					SD: 61 (47.0, 71.0)	SD: 57.0			SD: 10/286	SD: 4/286	SD:117/286
Perepu et al. (23)	U.S.	ID vs. SD	Randomized clinical trial, multicenter	176	ID: 65.0 (24.0, 86.0)	ID: 54.0	255 days	60.80	ID: 11/87	ID: 2/87	ID: 13/87
					SD: 63.5 (30.0, 85.0)	SD: 58.1			SD: 6/86	SD: 2/86	SD: 2/86
Alrashed et al. (24)	Saudi Arabian	TD vs. SD, ID vs. SD	Randomized clinical trial, multicenter	551	TD: 55.6 ± 13.12	TD: 79.9	17 months	100	TD: 43/179	TD: 18/179	TD: 104/179
					ID: 56.4 ± 13.79	ID: 78.3			ID: 28/180	ID: 6/180	ID: 93/180
					SD: 59.2 ± 14.98	SD: 68.2			SD: 25/192	SD: 6/192	SD: 112/192
Bikdeli et al. (25)	U.S.	ID vs. SD	Randomized clinical trial, multicenter	562	ID: 62 (51.0, 70.7)	ID: 58.7	113 days	100	ID: 9/276	ID: 7/276	ID: 127/276
					SD: 61 (47.0, 71.0)	SD: 57.0			SD: 10/286	SD: 4/286	SD: 123/286
Lawler et al. (26)	9 countries	TD vs. SD	Randomized clinical trial, multicenter	2,231	TD: 59.0 ± 14.1	TD: 60.4	276 days	2.02	TD: 13/1,180	TD: 22/1,180	TD: 86/1,180
					SD: 58.8 ± 13.9	SD: 56.9			SD: 22/1,046	SD: 9/1,047	SD: 86/1,046
Goligher et al. (27)	3 international adaptive platform	TD vs. SD	Randomized clinical trial, multicenter	1,103	TD: 60.4 ± 13.1	TD: 72.2	242 days	100	TD: 38/530	TD: 20/529	TD: 199/534
					SD: 61.7 ± 12.5	SD: 67.9			SD: 62/559	SD: 13/562	SD: 200/564
Lemos et al. (28)	Brazil	TD vs. SD	Randomized clinical trial, single center	20	TD: 55.0 ± 10.0	TD: 90.0	4 months	100	TD: 2/10	TD: 0/10	TD: 2/10
					SD: 58.0 ± 16.0	SD: 70.0			SD: 2/10	SD: 0/10	SD: 5/10
Lopes et al. (29)	Brazil	TD vs. SD	Randomized clinical trial, multicenter	615	TD: 56.7 ± 14.1	TD: 61.7	247 days	6.50	TD: 11/310	TD: 10/310	TD: 35/310
					D: 56.5 ± 14.5	SD: 57.9			SD: 18/304	SD: 4/304	SD: 23/304
Sholzberg et al. (30)	6 countries	TD vs. SD	Randomized clinical trial, multicenter	465	TD: 60.4 ± 14.1	TD: 53.9	318 days	16.13	TD: 2/228	TD: 2/228	TD: 4/228
					SD: 59.6 ± 15.5	SD: 59.5			SD: 6/237	SD: 4/237	SD: 18/237
Muñoz-Rivas et al. (31)	Spain	TD vs. SD, ID vs. SD	Randomized clinical trial, multicenter	300	TD: 58.5 ± 14.4	TD: 60.2	241 days	7.67	TD: 2/103	TD: 3/103	TD: 2/103
					ID: 56.5 ± 14.1	ID: 62.6			ID: 2/91	ID: 3/91	ID: 3/91
					SD: 54.1 ± 15.0	SD: 59.4			SD: 4/106	SD: 4/106	SD: 2/106
Matli et al. (32)	Lebanon	TD vs. SD	Retrospective cohort, single center	82	TD: 62.55 ± 15.80	TD: 67.7	10 months	20.73	TD: 9/31	TD: 2/31	TD: 7/31
					SD: 59.69 ± 17.04	SD: 58.8			SD: 5/51	SD: 5/51	SD: 5/51
Bohula et al. (33)	U.S.	TD vs. SD	Randomized clinical trial, multicenter	382	TD: 59 (50, 70)	TD: 61.8	574 days	100	TD: 18/191	TD: 4/191	TD: 1/191
					SD: 62 (51, 68)	SD: 56.5			SD: 28/191	SD: 1/191	SD: 1/191
Marcos-Jubilar et al. (34)	Spain	TD vs. SD	Randomized clinical trial, multicenter	65	TD: 63.0 ± 13.7	TD: 53.1	8 months	20.00	TD: 0/32	TD: 0/32	TD: 2/32
					SD: 62.3 ± 12.2	SD: 72.7			SD: 2/33	SD: 0/33	SD: 1/33
Sholzberg et al. (35)	U.S.	TD vs. SD	Randomized clinical trial, multicenter	257	67	54	30 days	33.07	NA	TD: 6/130	TD: 25/130
										SD: 2/127	SD: 32/127
Myers et al. (36)	U.S.	TD vs. SD, ID vs. SD	Retrospective cohort, multicenter	17,130	TD: 62.4 ± 14.4	TD: 65.3	305 days	29.45	TD: 56/1,721	TD: 119/1,721	TD: 453/1,721
					ID: 56.8 ± 14.7	ID: 60.1			ID: 58/6,754	ID: 160/6,754	ID: 649/6,754
					SD: 59.8 ± 16.0	SD: 55.9			SD: 99/8,655	SD: 189/8,655	SD:968/8,655
Gabara et al. (10)	Spain	TD vs. SD, ID vs. SD	Randomized clinical trial, single center	201	TD: 68.1 ± 9.6	TD: 72.4	2 months	100	TD: 6/29	TD: 9/29	TD: 8/29
					ID: 62.4 ± 12.5	ID: 69.1			ID: 21/94	ID: 14/94	ID: 17/94
					SD: 59.5 ± 13.6	SD: 71.8			SD: 14/78	SD: 4/78	SD: 17/78
Llitjos et al. (12)	France	TD vs. SD	Retrospective cohort, multicenter	26	TD: 67.5 ± 5.7	TD: 77.8	23 days	100	TD: 10/18	NA	TD: 2/18
					SD: 68 ± 6.9	SD: 75.0			SD: 8/8		SD: 1/8
Hoogenboom et al. (37)	U.S.	TD vs. SD	Retrospective cohort, single center	311	TD: 63.0 (53.0, 72.0)	TD: 71.9	144 days	100	TD: 12/153	NA	TD: 73/153
					SD: 56.0 (48.0, 67.0)	SD: 66.5			SD: 3/158		SD: 44/158

NA, not available; VTE, venous thromboembolism; TD, therapeutic dose; ID, intermediate dose; SD, standard prophylactic dose. Severity case was defined as (i) need for intensive care unit admission (ii) need for mechanical ventilation with tracheal intubation (iii) CT showing severe lung invasion (iv) acute respiratory failure (v) death (vi) severe and or critical on the basis of the WHO novel grading of the severity of COVID-19. The presence of either of the above items was classified as severe case.

The 19 literature had a total of 25,289 COVID-19 patients, including 12,549 who received standard-dose prophylactic anticoagulation, 7,758 who received intermediate-dose prophylactic anticoagulation, and 4,982 received therapeutic-dose prophylactic anticoagulation. The total weighted mean age was 58.27 years. The weighted mean age of standard-dose, intermediate-dose, and therapeutic-dose prophylactic anticoagulation was 59.84 years, 57.32 years, and 60.74 years, respectively. Males accounted for 59.58% of the total study population. The weighted proportion of males in standard-dose, intermediate-dose, and therapeutic-dose prophylactic anticoagulation was 57.22%, 60.49%, and 64.09%, respectively. The ICU admission rate was 50.96%. There were 7 studies in the United States, 1 in Saudi Arabia, 1 in Iran, 2 in Brazil, 1 in France, 3 in Spain, 1 in Lebanon, and 3 in multinational cooperative program. The sample size ranged from 20 to 17,130, and the median or mean follow-up time ranged from 30 days to 12 months.

**Figure 2:**
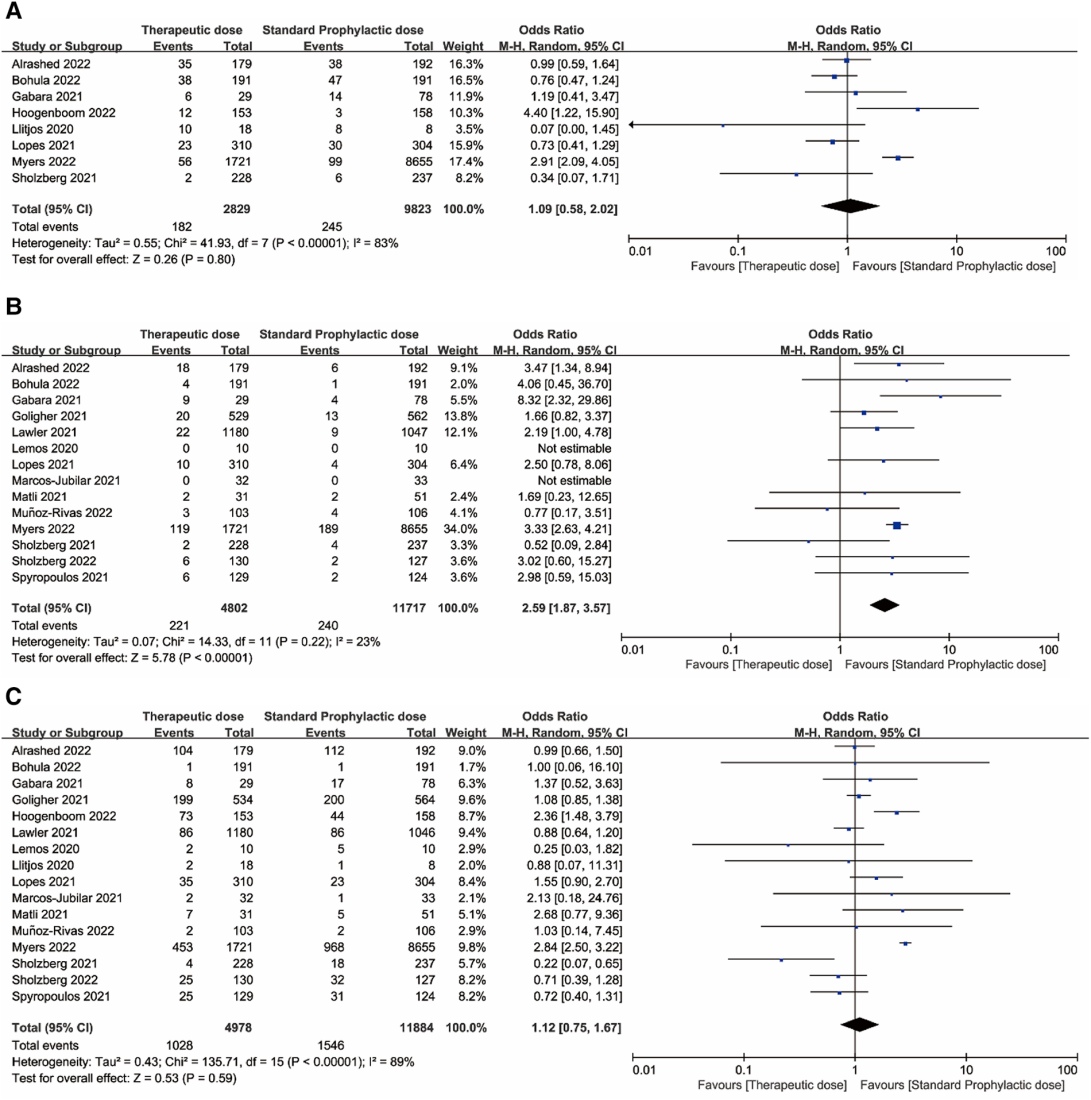
(**A**) Venous thromboembolism. (**B**) Major bleeding. (**C**) Mortality. Forest plots comparing effects of therapeutic-dose anticoagulation with standard-dose prophylactic anticoagulation regimen.

**Figure 3:**
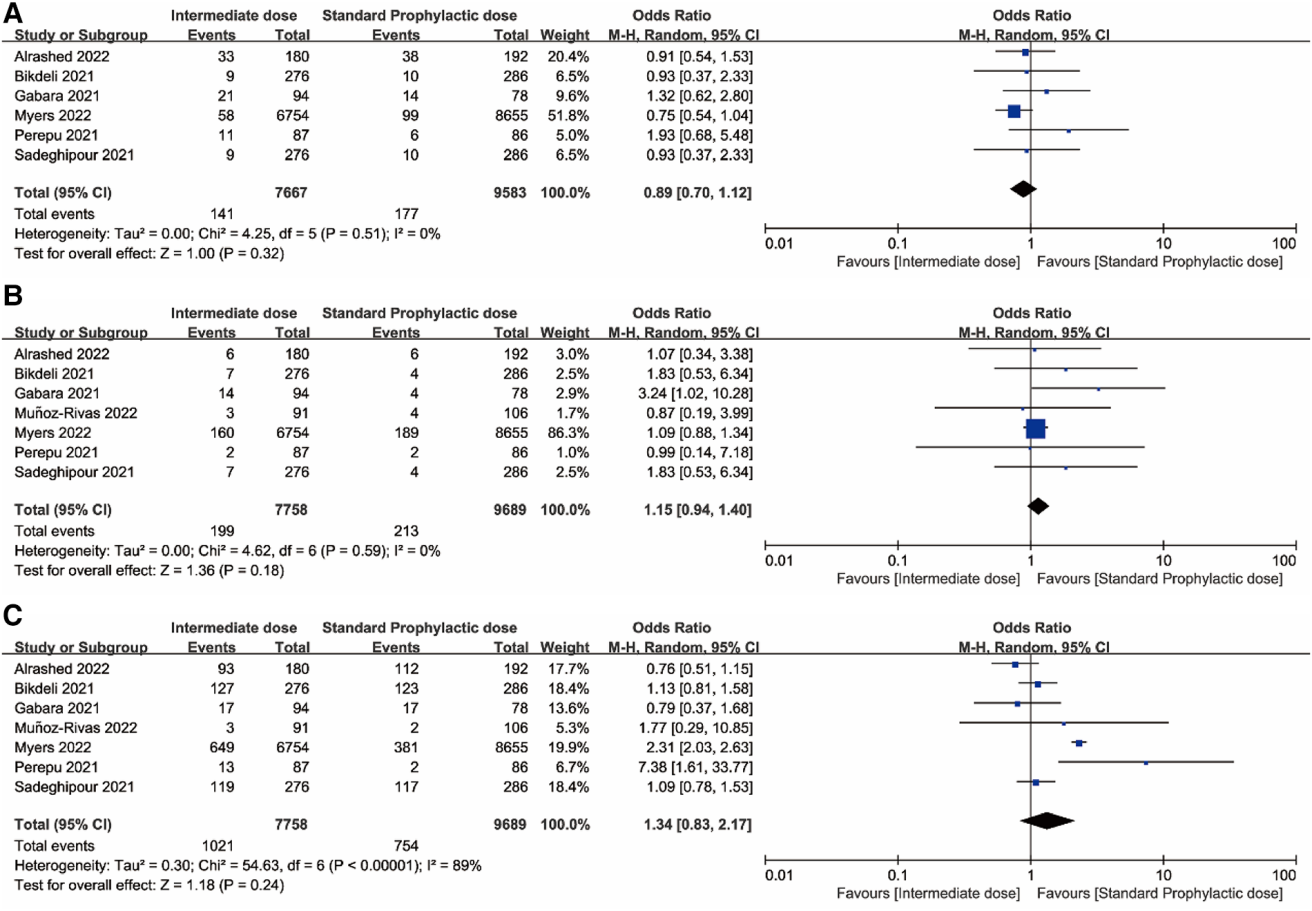
(**A**) Venous thromboembolism. (**B**) Major bleeding. (**C**) Mortality. Forest plots comparing effects of intermediate-dose anticoagulation versus standard-dose prophylactic anticoagulation regimen.

Figures 2, 3 displayed forest plots and the results were as follows. Compared with standard-dose prophylactic anticoagulation, results of therapeutic-dose prophylactic anticoagulation were: (1) VTE: I^2 ^= 83%, *P *= 0.80, OR = 1.09 (95% CI: 0.58, 2.02); (2) Major bleeding: I^2 ^= 23%, *P *< 0.00001, OR = 2.59 (95% CI: 1.87, 3.57); (3) Mortality: I^2 ^= 89%, *P *= 0.59, OR = 1.12 (95% CI: 0.75, 1.67). Compared with standard-dose prophylactic anticoagulation, results of intermediate-dose prophylactic anticoagulation were: (1) VTE: I^2 ^= 0%, *P *= 0.32, OR = 0.89 (95% CI: 0.70, 1.12); (2) Major bleeding: I^2 ^= 0%, *P *= 0.18, OR = 1.15 (95% CI: 0.94, 1.40); (3) Mortality: I^2 ^= 89%, *P *= 0.24, OR = 1.34 (95% CI: 0.83, 2.17).

**Figure 4:**
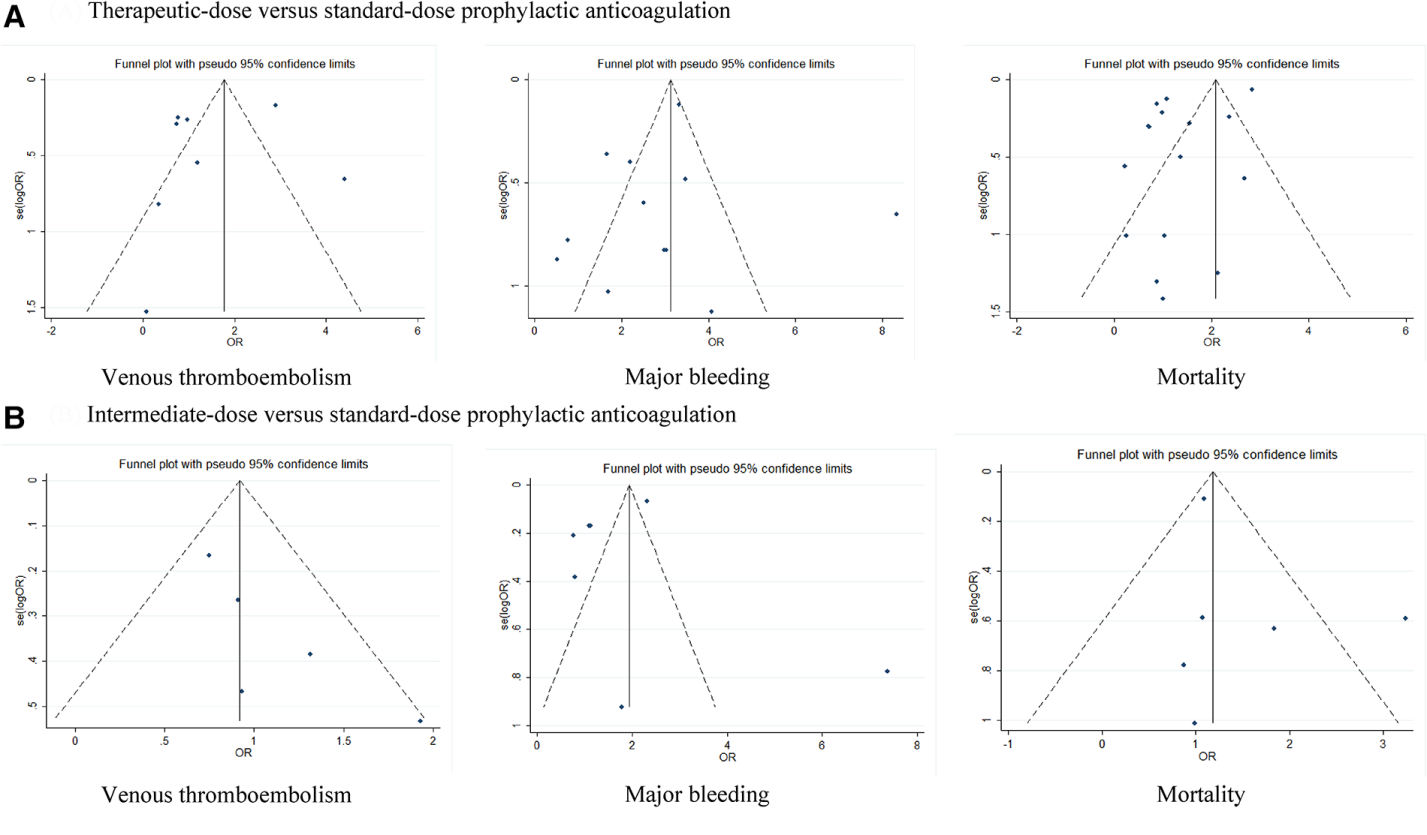
Funnel plots: effects of therapeutic-dose, intermediate-dose anticoagulation versus standard-dose prophylactic anticoagulation regimen. (**A**) Therapeutic-dose versus standard-dose prophylactic anticoagulation. (**B**) Intermediate-dose versus standard-dose prophylactic anticoagulation.

**Figure 5:**
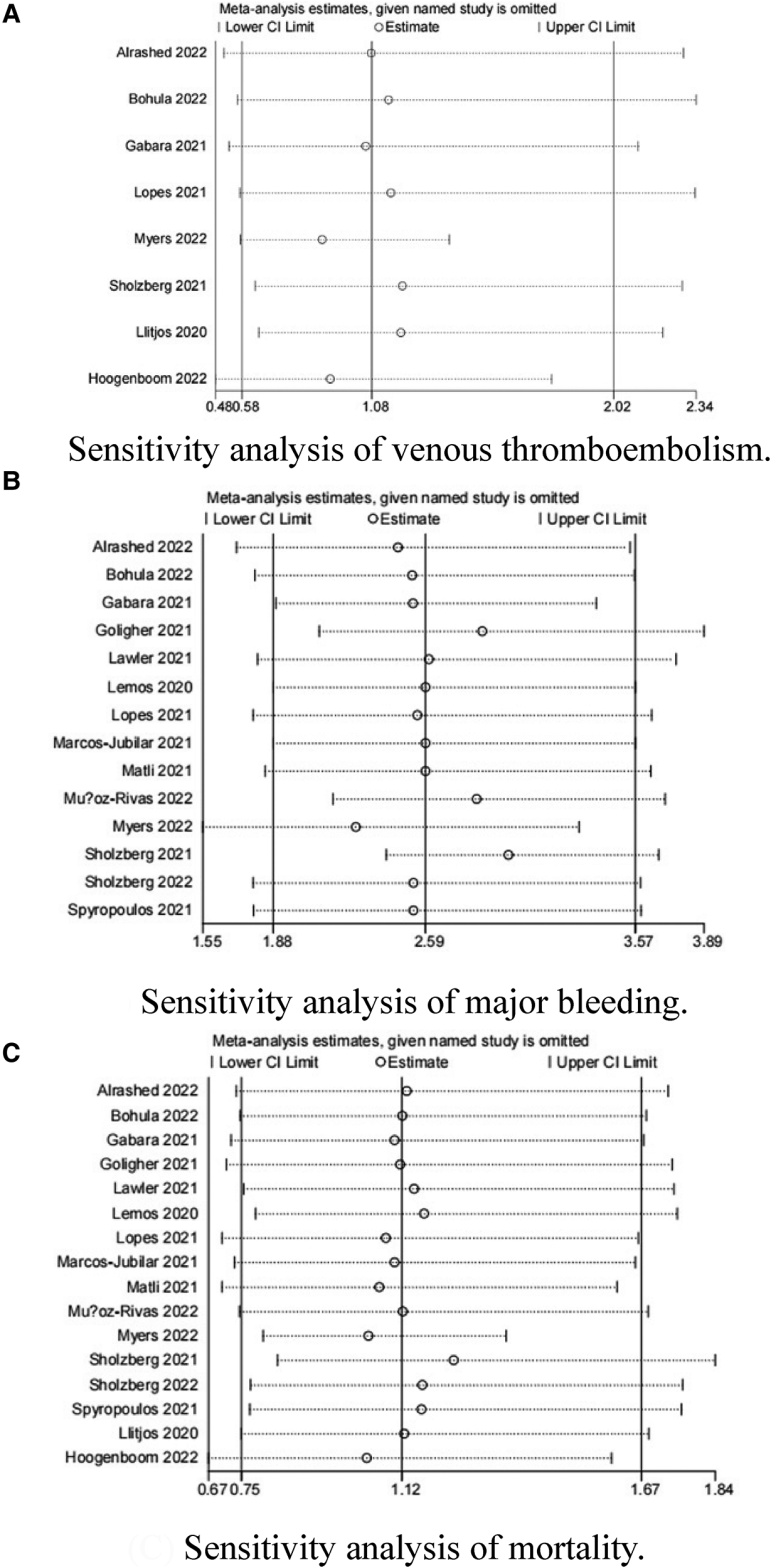
Sensitivity analysis: therapeutic-dose anticoagulation versus standard-dose prophylactic anticoagulation regimen. (**A**) Sensitivity analysis of venous thromboembolism. (**B**) Sensitivity analysis of major bleeding. (**C**) Sensitivity analysis of mortality.

**Figure 6:**
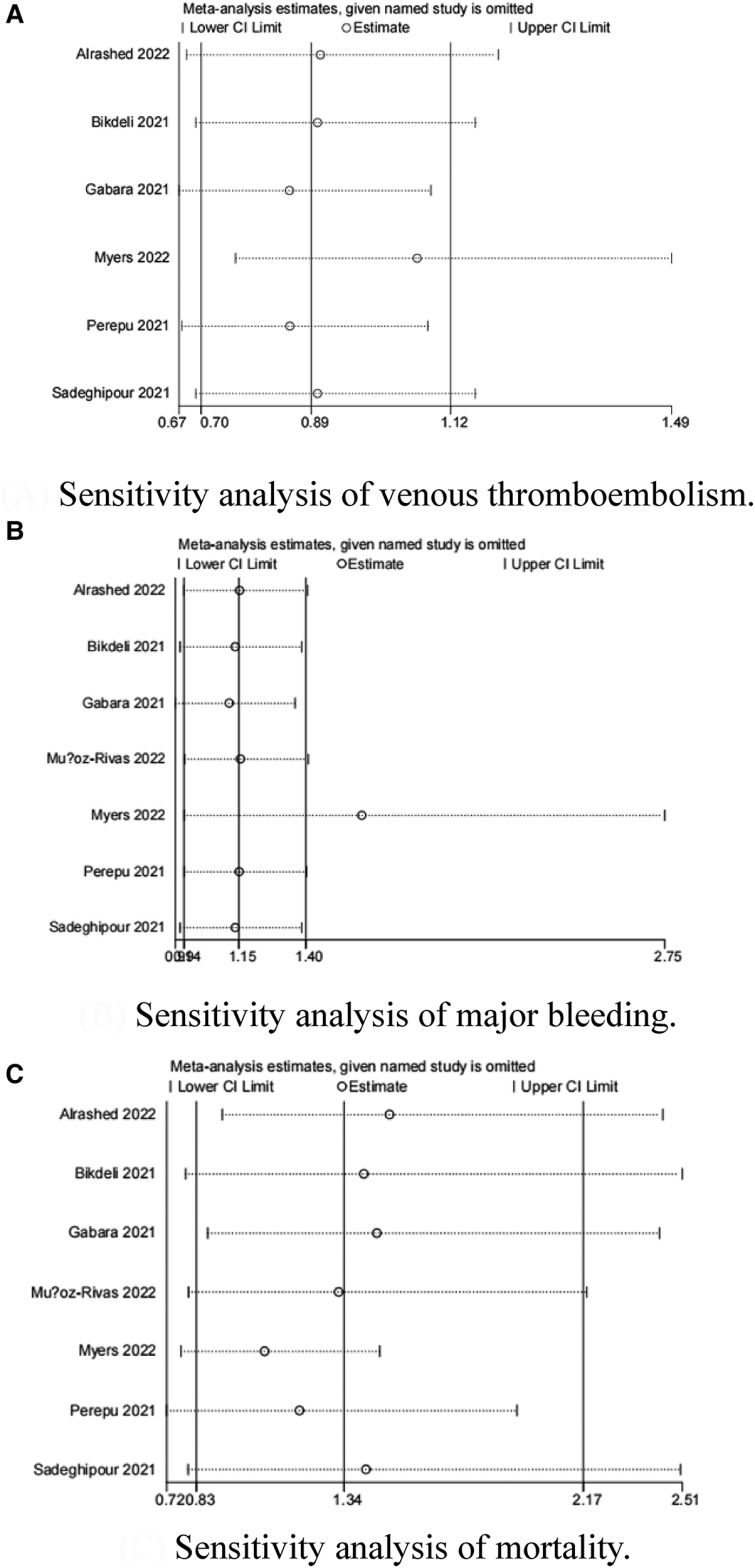
Sensitivity analysis: intermediate-dose anticoagulation versus standard-dose prophylactic anticoagulation regimen. (**A**) Sensitivity analysis of venous thromboembolism. (**B**) Sensitivity analysis of major bleeding. (**C**) Sensitivity analysis of mortality.

Because the I^2^ of some forest plots was greater than 50%, we continued to conduct funnel plots (Figure 4) and sensitivity analysis (Figures 5, 6). Results of pairwise comparison between therapeutic-dose and standard-dose prophylactic anticoagulation were: (1) VTE: Bohula et al. (33), Gabara et al. (10), Lopes et al. (29), Llitjos et al. (12), and Sholzberg et al. (30) had factors that might affect the results; (2) Major bleeding: Bohula et al. (33), Lopes et al. (29), Matli et al. (32), Marcos-Jubilar et al. (34), Sholzberg et al. (30), Sholzberg et al. (35), and Spyropoulos et al. (16) had factors that might affect the results; (3) Mortality: Bohula et al. (33), Gabara et al. (10), Goligher et al. (27), Lawler et al. (26), Lemos et al. (28), Lopes et al. (29), Marcos-Jubilar et al. (34), Muñoz-Rivas et al. (31), Sholzberg et al. (35), and Spyropoulos et al. (16) had factors that might affect the results. Results of pairwise comparison between therapeutic-dose and standard-dose prophylactic anticoagulation were: (1) VTE: combined with I^2 ^= 0.0%, fewer factors might affect the results; (2) Major bleeding: combined with I^2 ^= 0.0%, Gabara et al. (10) had factors that might affect the results; (3) Mortality: Bikdeli et al. (25), Gabara et al. (10), Muñoz-Rivas et al. (31), and Sadeghipour et al. (22) had factors that might affect the results.

In addition, we also developed NOS for the evaluation of literature quality (Table 3), indicating that the selected articles were of good quality.

**Table 3:** Literature quality assessment by the Newcastle-Ottawa scale.

Author (publication year)	Adequate definition of cases	Representativeness of the cases	Selection of controls	Definition of controls	Control for important factor	Ascertainment of exposure	Same method of ascertainment for cases and controls	Non-response rate	score
Spyropoulos et al. (16)	★	★	★	★	★★	★	★	★	9
Sadeghipour et al. (22)	★	★	★	★	★☆	★	★	★	8
Perepu et al. (23)	★	★	★	★	★☆	★	★	★	8
Alrashed et al. (24)	★	☆	★	★	★☆	★	★	★	7
Bikdeli et al. (25)	★	★	★	★	★☆	★	★	★	8
Lawler et al. (26)	★	★	★	★	★★	★	★	★	9
Goligher et al. (27)	★	★	★	★	★★	★	★	★	9
Lemos et al. (28)	★	☆	★	★	★☆	★	★	★	7
Lopes et al. (29)	★	★	★	★	★★	★	★	★	9
Sholzberg et al. (30)	★	☆	★	★	★☆	★	★	☆	6
Muñoz-Rivas et al. (31)	★	★	★	★	★☆	★	★	★	8
Matli et al. (32)	★	☆	★	★	★☆	★	★	☆	6
Bohula et al. (33)	★	★	★	★	★☆	★	★	★	8
Marcos-Jubilar et al. (34)	★	☆	★	★	★☆	★	★	★	7
Sholzberg et al. (35)	★	★	★	★	★☆	★	★	★	8
Myers et al. (36)	★	★	★	★	★☆	★	★	☆	7
Gabara et al. (10)	★	☆	★	★	★☆	★	★	☆	6
Llitjos et al. (12)	★	★	★	★	★☆	★	★	★	8
Hoogenboom et al. (37)	★	☆	★	★	★☆	★	★	☆	6

★, Means one point; ☆, no point.

Based on the above analysis, we further con ducted a subgroup analysis to identify the source of heterogeneity in terms of elders (65 years), gender, study duration, study design, and ICU admission rate. See Table 4 for the results of the subgroup analysis.

**Table 4:** Subgroup and sensitivity analyses for the primary outcomes.

	No. of studies	Odds ratio	95% Cl	*p*	I^2^	Q statistic	*P* for subgroup
Therapeutic dose vs. standard dose							
VTE							
Age							
Age ≥65 years old	2	0.41	(0.03, 6.56)	0.53	69%	3.22	0.43
Age <65 years old	6	1.29	(0.62, 2.70)	0.49	85%	34.44	
ICU admission							
ICU admission rate = 100%	5	1.23	(0.52, 2.91)	0.63	75%	15.79	0.75
ICU admission rate ≠ 100%	3	0.94	(0.23, 3.88)	0.94	90%	19.76	
Duration							
Duration ≥180 days	5	1.07	(0.48, 2.39)	0.86	88%	32.70	0.94
Duration <180 days	3	1.15	(0.22, 6.17)	0.87	71%	6.88	
Sex							
Male% ≥70%	4	1.66	(0.68, 4.07)	0.27	58%	7.17	0.36
Male% <70%	4	0.85	(0.28, 2.59)	0.77	91%	32.36	
Study type							
Randomized clinical trials	6	1.10	(0.54, 2.23)	0.79	85%	33.16	0.83
Retrospective cohort	2	0.70	(0.01, 43.89)	0.87	85%	6.52	
Severity							
Critical	5	0.95	(0.70, 1.30)	0.76	57%	9.29	0.22
Non critical	1	0.34	(0.07, 1.71)	0.19	54%	NA	
Major bleeding event							
Age							
Age ≥65 years old	3	4.73	(2.02, 11.09)	**<0**.**001**	0%	1.38	0.13
Age <65 years old	11	2.32	(1.60, 3.35)	**<0**.**001**	32%	11.76	
ICU admission							
ICU admission rate = 100%	5	3.19	(1.53, 6.63)	**0**.**002**	42%	5.19	0.52
ICU admission rate ≠ 100%	9	2.42	(1.62, 3.60)	**<0**.**001**	23%	9.07	
Duration							
Duration ≥180 days	11	2.41	(1.73, 3.36)	**<0**.**001**	23%	11.76	0.11
Duration <180 days	3	5.65	(2.07, 15.39)	**<0**.**001**	0%	0.94	
Sex							
Male% ≥70%	5	3.21	(1.34, 7.71)	**0**.**01**	61%	5.07	0.67
Male% <70%	9	2.61	(1.86, 3.65)	**<0**.**001**	12%	9.13	
Study type							
Randomized clinical trials	13	2.59	(1.84. 3.65)	**<0**.**001**	29%	14.03	0.68
Retrospective cohort	1	1.69	(0.23, 12.65)	0.61	NA	NA	
Severity							
Critical	5	2.78	(1.73, 4.47)	**< 0.001**	23%	5.20	0.12
Non critical	5	1.49	(0.81, 2.76)	0.20	37%	3.16	
Mortality group							
Age							
Age ≥65 years old	4	0.79	(0.54, 1.16)	0.23	0%	1.44	0.16
Age <65 years old	12	1.22	(0.77, 1.93)	0.40	90%	114.33	
ICU admission							
ICU admission rate = 100%	7	1.24	(0.85, 1.79)	0.26	50%	12.03	0.72
ICU admission rate ≠ 100%	9	1.08	(0.58, 2.01)	0.80	92%	96.64	
Duration							
Duration ≥180 days	11	1.13	(0.69, 1.84)	0.64	92%	119.84	0.94
Duration <180 days	5	1.09	(0.51, 2.32)	0.83	69%	12.90	
Sex							
Male% ≥70%	7	1.26	(0.87, 1.82)	0.23	51%	12.23	0.62
Male% <70%	9	1.05	(0.56,1.95)	0.88	92%	96.93	
Study type							
Randomized clinical trials	13	0.98	(0.62, 1.55)	0.93	91%	133.97	**0**.**01**
Retrospective cohort	3	2.33	(1.51, 3.60)	**<0**.**001**	0%	0.62	
Severity							
Critical	7	1.16	(0.97, 1.38)	0.11	52%	12.51	**0**.**02**
Noncritical	5	1.03	(0.57, 1.01)	0.06	49%	7.77	
Intermediate dose vs. standard dose							
VTE							
Age							
Age ≥65 years old	2	1.50	(0.81, 2.77)	0.19	0%	0.34	0.10
Age <65 years old	4	0.86	(0.66, 1.11)	0.70	5%	2.21	
ICU admission							
ICU admission rate = 100%	4	1.14	(0.78, 1.66)	0.50	0%	0.58	0.87
ICU admission rate ≠ 100%	2	1.05	(0.43, 2.56)	0.91	65%	2.89	
Duration							
Duration ≥180 days	3	1.04	(0.63, 1.72)	0.88	54%	4.39	0.92
Duration <180 days	3	1.08	(0.66, 1.76)	0.77	0%	0.46	
Sex							
Male% ≥70%	2	1.26	(0.80, 2.00)	0.32	0%	0.02	0.70
Male% <70%	4	0.84	(0.63, 1.11)	0.22	1%	3.02	
Severity							
Critical	4	1.00	(0.70, 1.42)	0.98	0%	0.68	0.24
Noncritical	1	1.07	(0.77, 1.50)	0.22	NA	NA	
Major Bleeding group							
Age							
Age ≥65 years old	2	2.38	(0.86, 6.60)	0.10	3%	1.03	0.15
Age <65 years old	5	1.11	(0.91, 1.36)	0.30	0%	1.40	
ICU admission							
ICU admission rate = 100%	4	1.85	(1.02, 3.35)	**0**.**04**	0%	1.78	0.10
ICU admission rate ≠ 100%	3	1.08	(0.88, 1.33)	0.47	0%	0.09	
Duration							
Duration ≥180 days	4	1.08	(0.88, 1.33)	0.46	0%	0.09	0.05
Duration <180 days	3	2.26	(1.12, 4.54)	**0**.**02**	0%	0.59	
Sex							
Male% ≥70%	2	1.86	(0.63, 5.52)	0.26	44%	1.78	0.36
Male% <70%	5	1.11	(0.91, 1.36)	0.31	0%	1.41	
Severity							
Critical	4	1.89	(1.05, 3.39)	**0**.**03**	0%	1.78	0.29
Noncritical	2	0.91	(0.27, 3.05)	0.06	0%	0.01	
Mortality group							
Age							
Age ≥65 years old	2	2.19	(0.24, 20.16)	0.49	85%	6.90	0.64
Age <65 years old	5	1.27	(0.75, 2.15)	0.38	91%	0.38	
ICU admission							
ICU admission rate = 100%	4	1.00	(0.82, 1.21)	0.96	0%	2.83	**<0**.**001**
ICU admission rate ≠ 100%	3	2.49	(1.59, 3.90)	**<0**.**001**	14%	2.31	
Duration							
Duration ≥180 days	4	1.87	(0.79, 4.44)	0.15	89%	28.11	0.22
Duration <180 days	3	1.08	(0.86, 1.35)	0.51	0%	0.73	
Sex							
Male% ≥70%	2	0.77	(0.54, 1.10)	0.15	0%	0.01	**0**.**02**
Male% <70%	5	1.68	(1.00, 2.83)	0.05	87%	31.08	
Severity							
Critical	4	1.00	(0.82, 1.21)	0.96	0%	2.83	**0**.**01**
Noncritical	2	4.51	(1.48, 13.80)	**0.008**	30%	1.42	

Bold values indicate significant *p*-values < 0.05.

The main purpose of network meta-analysis was to compare the difference between therapeutic-dose and intermediate-dose prophylactic anticoagulation. Further, ranking and surface under the cumulative ranking (SUCRA) probabilities were performed to carry out the recommended order of the three doses after evaluation of the incidence of VTE, major bleeding and mortality.

**Figure 7:**
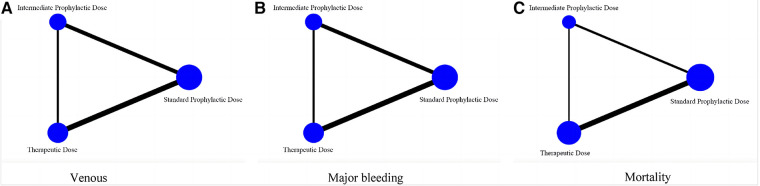
Network meta-analysis diagram comparing efficacy and safety of three prophylactic anticoagulation regimen for COVID-19 patients. (**A**) Venous thromboembolism. (**B**) Major bleeding. (**C**) Mortality.

As shown in Figure 7, this network meta-analysis had a closed-loop structure, so its results were to merge the direct and indirect comparisons and make decisions accordingly.

**Figure 8:**
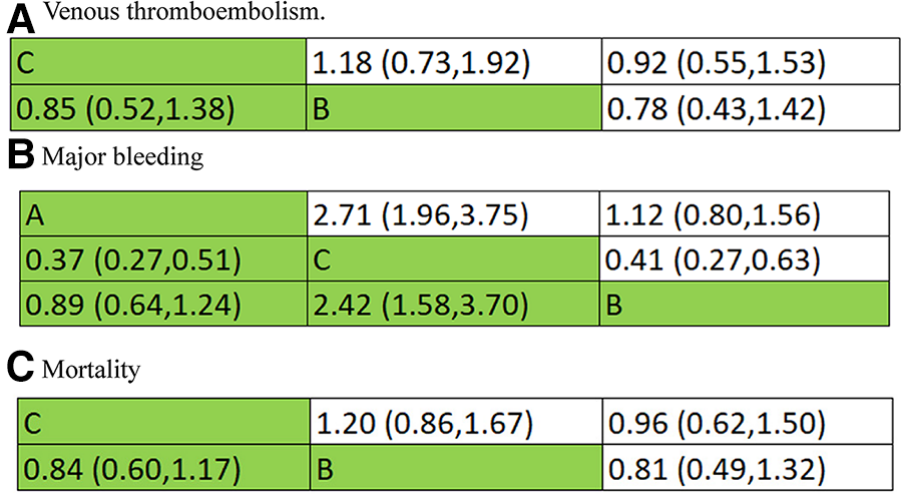
Inverted triangle plot of network meta-analysis: therapeutic-dose versus intermediate-dose prophylactic anticoagulation regimen. (**A**) Venous thromboembolism. (**B**) Major bleeding. (**C**) Mortality.

Combined with the inverted triangle plot (Figure 8), compared with intermediate-dose prophylactic anticoagulation, OR and 95%CI of VTE, major bleeding and mortality in therapeutic-dose prophylactic anticoagulation was 0.85 (95% CI: 0.52, 1.38), 2.42 (95% CI: 1.58, 3.70) and 0.84 (95% CI: 0.60, 1.17).

**Figure 9:**
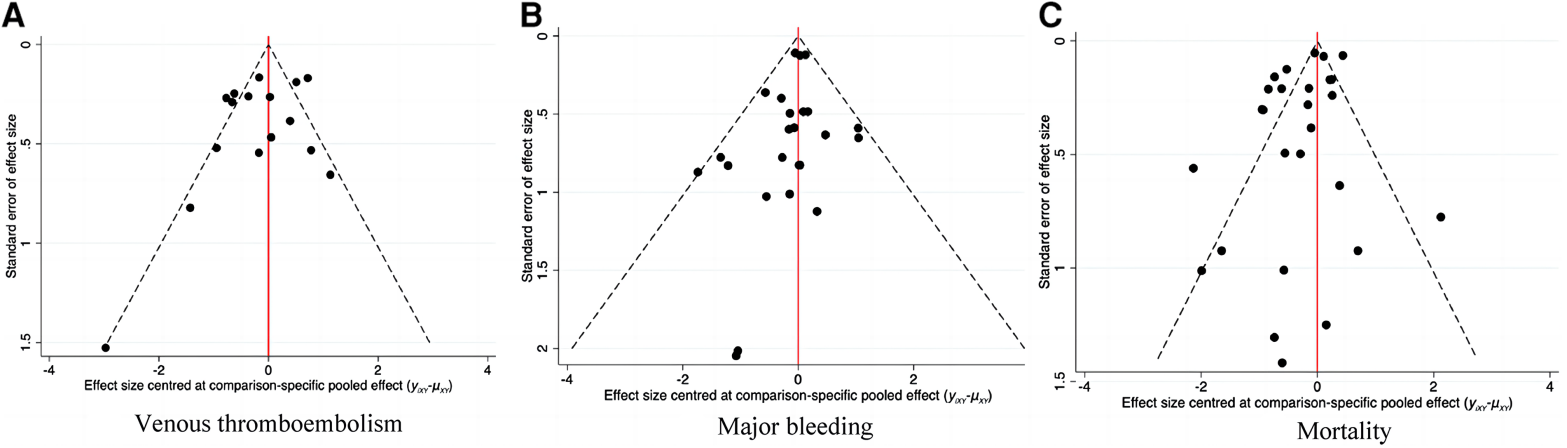
Corrected funnel plot of network meta-analysis. (**A**) Venous thromboembolism. (**B**) Major bleeding. (**C**) Mortality.

The adjusted funnel plot in Figure 9 pointed out no evidence of publication bias in our included articles.

**Figure 10:**
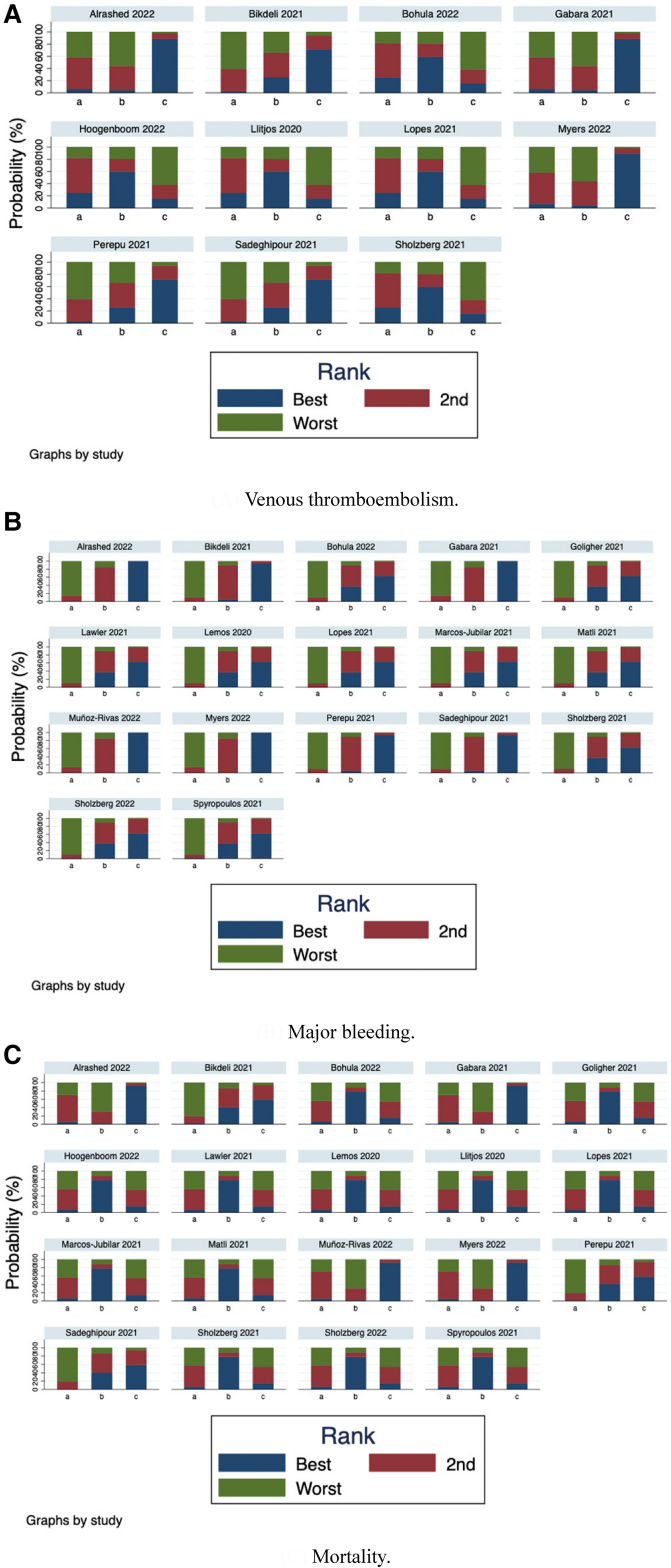
Rank-order plot of network meta-analysis. (**A**) Venous thromboembolism. (**B**) Major bleeding. (**C**) Mortality.

**Figure 11:**
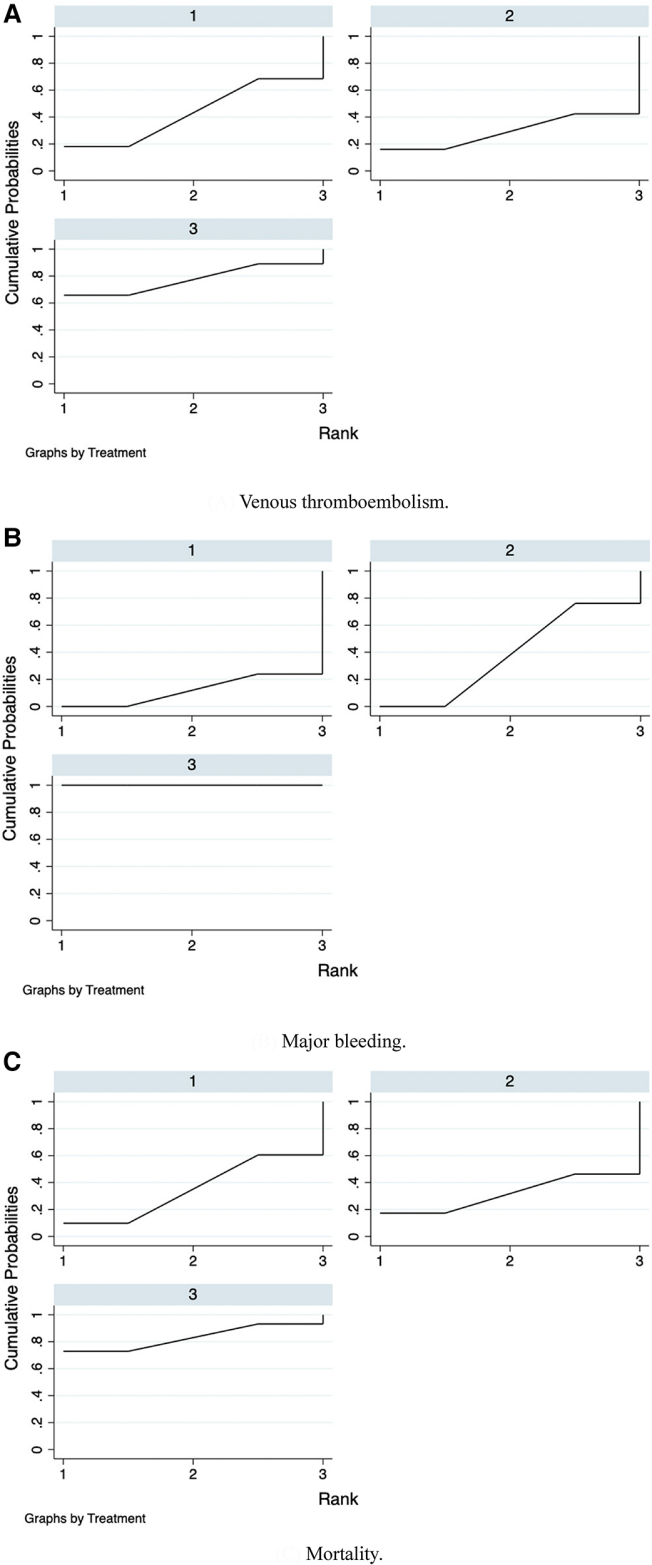
SUCRA plots of network meta-analysis. (**A**) Venous thromboembolism. (**B**) Major bleeding. (**C**) Mortality.

Furthermore, we ranked the impact of three doses of prophylactic anticoagulation on VTE, major bleeding, and mortality in patients with COVID-19. Ranking and SUCRA were shown in Figures 10, 11, separately.

## 4 Discussion

SARS-CoV-2 infection can not only cause multiple organ damage (38), but greatly increase the risk of VTE. As early as the begin ning of the COVID-19 pandemic in 2020, studies from Wuhan, China, initially revealed that COVID-19 patients had a high risk of VTE (39), which was gradually confirmed with the outbreak all over the world. Marchandot et al. (40) summarized studies on hospitalized COVID-19 patients from different countries and found that the incidence of VTE in non-ICU and ICU patients was 3%–46% and 15%–85%, separately. Nopp et al. (41) conducted a meta-analysis of 66 clinical studies with 28,173 COVID-19 patients and indicated that the overall incidence of VTE was 14.1%. Among them, the incidence of VTE was 40.3% if lower extremity venous color Doppler ultrasound screening was used, and 9.5% if no ultrasound screening was used. Stals MAM et al. (42) analyzed 3 ho spitals in the Netherlands and reported that the incidence of VTE in hospitalized COVID-19 patients was 18.7%, while that in hospitalized patients with influenza from 2013 to 2018 was only 1.04%. Although the incidence of VTE varies from study to study, there is a consensus that the risk of VTE remains higher in COVID-19 patients, and the more severe the disease, the higher the risk (43).

On the other hand, the prognosis of COVID-19 patients tends to be worse if VTE occurs. A study (44) from Wuhan, China, enrolled 143 COVID-19 cases in the ICU and noted that compared with patients without DVT, the mortality of those who had comorbid DVT was significantly higher (34.8% vs.11.7%, *P *= 0.001). Kollias A et al. (45) developed a meta-analysis of more than 6,000 patients and revealed that the incidence of PE and DVT in hospitalized patients with COVID-19 was 32% and 27%, respectively, and the risk of death was twice higher if VTE was accompanied. Even if VTE is not the direct cause of death, it may be an important cause. Lax et al. (46) from Australia analyzed an autopsy study on 11 patients who died of COVID-19 and proved that all patients had comorbid PE (46). Wichmann et al. (8) published an autopsy report on 12 patients who died of COVID-19, showing that 58% had DVT and 33% died of PE rather than COVID-19. Therefore, early scientific and reasonable prevention and treatment of VTE is essential to improve the prognosis of COVID-19. However, since VTE and COVID-19 share many vital signs and clinical symptoms, it becomes difficult to identify in the early stage, so prophylactic anticoagulation emerges as the times require. With the growing evidence on the association between prophylactic anticoagulation and lower mortality among COVID-19 patients, the International Society on Thrombosis and Hemostasis (ISTH) (47) and American College of Clinical Pharma (ACCP) (48, 49) have issued relevant clinical guidelines or expert consensus and recommended standard-dose prophylactic anticoagulation for all hospitalized COVID-19 patients if there is no contraindication. In clinical practice, however, VTE still occurs in some hospitalized cases receiving standard-dose prophylactic anticoagulation (12, 47, 50). Considering the high incidence of COVID-19 combined with VTE and the high mortality due to disease progression, prophylactic anticoagulation with higher doses than standard has been carried out in many hospitals (49, 51), which may place patients at higher risk for major bleeding (13, 15, 52). Controversy exists regarding which thromboprophylaxis treatment can achieve better clinical benefits in hospitalized patients with COVID-19.

Benefits from the use of standard-dose prophylactic anticoagulation in patients with COVID-19 remain controversial. Almohareb et al. (9) and Gabara et al. (10) both supported standard-dose prophylactic anticoagulation in COVID-19 patients, the former confirmed that increasing dose over the standard was not associated with reduced mortality, and the latter implied that the use of intermediate-dose and therapeutic-dose prophylactic anticoagulation seemed to have a higher risk of bleeding in critical COVID-19 cases. Cohen et al. (11) identified that compared with treatment-dose anticoagulation, prophylactic-dose anticoagulation in COVID-19 patients could reduce VTE or mortality. On the contrary, Llitjos et al. (12) documented that the proportion of VTE was significantly higher in patients treated with standard-dose prophylactic anticoagulation than in other groups (i.e., intermediate-dose and therapeutic-dose).

The advantages of intermediate-dose prophylactic anticoagulation in patients with COVID-19 have not reached an agreement. Hamilton et al. (53) expressed that compared with standard-dose, intermediate-dose thromboprophylaxis in critical COVID-19 patients could have better levels of anti-FXa. A randomized clinical trial by Engelen et al. (14) displayed that in hospitalized patients with COVID-19, no additional symptomatic VTE occurred after the implementation of a systematic weight-adjusted thromboprophylaxis (prophylactic-dose in the general ward and intermediate-dose in ICU), and collateral DVT reduced. Al-Dorzi et al. (50) described the benefits of intermediate-dose enoxaparin in reducing VTE and mortality than standard-dose unfractionated heparin or enoxaparin in patients with severe COVID-19. However, the results of Aljuhani et al. (54) concluded that compared with the standard-dose prophylactic anticoagulation, intermediate-dose prophylactic anticoagulation was not associated with thrombosis or mortality in critical COVID-19, but increased risk of minor bleeding. Al-Abani et al. (13) performed ultrasound on COVID-19 patients in ICU with intermediate-dose prophylactic anticoagulation and illustrated that patients still had a high incidence of VTE and bleeding complications.

Consensus is needed regarding the efficacy of therapeutic-dose prophylactic anticoagulation in patients with COVID-19. Spyropoulos et al. (16) initiated a randomized clinical trial on COVID-19 patients and showed that therapeutic-doses of low-molecular-weight heparin could reduce thromboembolism and death. However, a prospective observational study by Kumar et al. (55) interpreted that the use of therapeutic-dose prophylactic anticoagulation in patients with COVID-19 did not reduce the incidence of VTE, but was associated with higher in-hospital mortality. In a retrospective study of 1,121 patients in 33 hospitals, Parks et al. (15) proposed that compared with other anticoagulation regimens, the incidence of VTE and bleeding in COVID-19 patients receiving therapeutic-dose anticoagulation was three times and five times higher, separately.

This meta-analysis included 19 studies published between January 1, 2020, and October 31, 2022. To our knowledge, this is the first meta-analysis conducting a *p* airwise comparison among three conventional prophylactic anticoagulations in the incidence of VTE, major bleeding, and mortality. This meta-analysis included 19 related studies with 25,289 COVID-19 patients, and the results showed that: (1) compared with standard-dose prophylactic anticoagulation, odds ratio (OR) and 95% confidence interval (CI) of VTE, major bleeding and mortality in therapeutic-dose prophylactic anticoagulation was 1.09 (95% CI: 0.58, 2.02), 2.59 (95% CI: 1.87, 3.57) and 1.12 (95% CI: 0.75, 1.67), respectively; (2) compared with standard-dose prophylactic anticoagulation, OR and 95%CI of VTE, major bleeding and mortality in intermediate-dose prophylactic anticoagulation was 0.89 (95% CI: 0.70, 1.12), 1.15 (95% CI: 0.94, 1.40) and 1.34 (95% CI: 0.83, 2.17), respectively; (3) compared with intermediate-dose prophylactic anticoagulation, OR and 95%CI of VTE, major bleeding and mortality in therapeutic-dose prophylactic anticoagulation was0.85 (95% CI: 0.52, 1.38), 2.42 (95% CI: 1.58, 3.70) and 0.84 (95% CI: 0.60, 1.17). The above results suggested that compared with COVID-19 patients receiving intermediate-dose or therapeutic-dose prophylactic anticoagulation, those who underwent standard-dose prophylactic anticoagulation had the lowest risk of bleeding events. In terms of VTE and mortality, no significant differences were found.

We further ranked the impact of the three doses of anticoagulation on VTE, major bleeding, and mortality in patients with COVID-19. Combining the results of ranking (Figure 10) and SUCRA (Figure 11), the order of probability of VTE events from high to low was: therapeutic-dose >> standard-dose > intermediate-dose. The order of probability of major bleeding events from high to low was: therapeutic-dose > intermediate-dose >> standard-dose. The order of probability of death events from high to low was: therapeutic-dose >> standard-dose > intermediate-dose. This ranking result further validated the previous results.

To verify the applicability of the above results, we conducted a subgroup analysis in terms of age, ICU admission rate, hospital stay, etc. It is worth noting that compared with the standard dose, although therapeutic-dose prophylactic anticoagulation increased the risk of major bleeding, it could significantly reduce VTE formation in patients over 65 years of age.

To sum up, consistent with ISTH guidelines and ACCP guidelines (49), we recommended a standard-dose rather than an above-standard dose (i.e., intermediate-dose or therapeutic-dose) for prophylactic anticoagulation in COVID-19 patients who received no anticoagulation therapy within 6 months before admission. Only for elderly COVID-19 patients with low bleeding risk and high VTE risk, we recommended therapeutic-dose prophylactic anticoagulation. In addition, the Caprini score is the most validated VTE risk assessment tool and has been used to evaluate the risk of VTE in approximately 5 million medical and surgical patients worldwide. Since COVID-19 patients are themselves at high risk for VTE, the revised Caprini Score has been tailored to the initial Caprini Score (2005 version), with the addition of a score for elevated D-dimer and a score for COVID-19 infections, specifically: asymptomatic infections are considered to be a 2-point score, symptomatic infections are considered to be a 3-point score, and symptomatic infections combined with elevated D-dimer are 5 points were considered (56). Based on this score, the risk of VTE in COVID-19 patients can be further evaluated and guide the application of clinical anticoagulation programs. Therefore, it is scientific and reasonable to provide standard-dose prophylactic anticoagulation for all hospitalized patients in a timely manner, to increase the dose individually for elderly patients with a high risk of VTE or acceptable risk of bleeding, as well as to adjust the dose according to the patient's weight and the disease progression. It is expected that there will be a higher level of evidence to verify our conclusion in the future.

There are some limitations in this study. First, the prevalence of thromboembolism in COVID-19 patients was likely to be underestimated. The possible reason was that the incidence of thrombotic events (e.g., PE, DVT, myocardial infarction, ischemic stroke, and other thromboembolism) diagnosed with routine clinical care was often less than that seen on computed tomography pulmonary angiography (CTPA). Second, we could only obtain a preliminary conclusion from our included articles on the comparison between therapeutic-dose or intermediate-dose and standard-dose prophylactic anticoagulation, as well as the comparison between therapeutic-dose and intermediate-dose prophylactic anticoagulation from the network meta-analysis. Meanwhile, considering the limited number of relevant clinical studies and the presence of heterogeneity, follow-up large-scale studies are required to further explore the safety and effectiveness of different treatments, so as to guide clinical practice and improve the disease status. Third, we encountered high statistical heterogeneity during the meta-analysis. Despite we conducted a prespecified sensitivity analysis, these failed to adequately explain such heterogeneity. This residual heterogeneity might derive from sources of variation between studies, most notably because of age, gender, race, lack of continuous registration, clinical measurements, nursing level, virus strains, and disease severity. Finally, most included studies were rated as having a moderate risk of bias, reflecting generally low methodological quality. The underlying explanations were the lack of control for confounders, inconsistent or unclear context in VTE evaluations, and possible selection bias due to the absence of continuous patient registration.

## 5 Conclusion

In terms of prevention and treatment of VTE, this study pointed out that COVID-19 patients in general could not benefit more from intermediate-dose or therapeutic-dose prophylactic anticoagulation than standard-dose prophylactic anticoagulation, while elderly COVID-19 patients with low bleeding risk and high VTE risk appeared to benefit more from therapeutic-dose prophylactic anticoagulation. Therefore, we suggested that individualized adjustment should be performed based on the standard-dose prophylactic anticoagulation according to the specific conditions of COVID-19 patients. At the same time, this meta-analysis further supported the expert consensus of ACCP guidelines that patients with COVID-19 should still receive standard-dose prophylactic anticoagulation, while non-critically ill patients with low bleeding risk might benefit from therapeutic-dose prophylactic anticoagulation. In summary, this meta-analysis only provided a preliminary conclusion for reference due to the objective limitations of different health service levels, types of strains, types, and doses of vaccines, presence of thromboprophylaxis, and thromboprophylaxis regimens. Further studies will still have positive clinical implications for COVID-19 patients.

## Data Availability

The original contributions presented in the study are included in the article/Supplementary Material, further inquiries can be directed to the corresponding authors.
